# How I report breast magnetic resonance imaging studies for breast cancer staging and screening

**DOI:** 10.1186/s40644-016-0078-0

**Published:** 2016-07-25

**Authors:** Sarah Vinnicombe

**Affiliations:** Cancer Research, Ninewells Hospital Medical School, University of Dundee, Dundee, DD1 9SY Scotland

**Keywords:** Magnetic resonance imaging, Breast cancer, Staging, BI-RADS, Screening

## Abstract

Magnetic resonance imaging (MRI) of the breast is the most sensitive imaging technique for the diagnosis and local staging of primary breast cancer and yet, despite the fact that it has been in use for 20 years, there is little evidence that its widespread uncritical adoption has had a positive impact on patient-related outcomes.

This has been attributed previously to the low specificity that might be expected with such a sensitive modality, but with modern techniques and protocols, the specificity and positive predictive value for malignancy can exceed that of breast ultrasound and mammography. A more likely explanation is that historically, clinicians have acted on MRI findings and altered surgical plans without prior histological confirmation. Furthermore, modern adjuvant therapy for breast cancer has improved so much that it has become a very tall order to show a an improvement in outcomes such as local recurrence rates.

In order to obtain clinically useful information, it is necessary to understand the strengths and weaknesses of the technique and the physiological processes reflected in breast MRI. An appropriate indication for the scan, proper patient preparation and good scan technique, with rigorous quality assurance, are all essential prerequisites for a diagnostically relevant study.

The use of recognised descriptors from a standardised lexicon is helpful, since assessment can then dictate subsequent recommendations for management, as in the American College of Radiology BI-RADS (Breast Imaging Reporting and Data System) lexicon (Morris et al., ACR BI-RADS® Atlas, Breast Imaging Reporting and Data System, 2013). It also enables audit of the service. However, perhaps the most critical factor in the generation of a meaningful report is for the reporting radiologist to have a thorough understanding of the clinical question and of the findings that will influence management. This has never been more important than at present, when we are in the throes of a remarkable paradigm shift in the treatment of both early stage and locally advanced breast cancer.

**Electronic supplementary material:**

The online version of this article (doi:10.1186/s40644-016-0078-0) contains supplementary material, which is available to authorized users.

## Background

The sensitivity of mammography for breast cancer detection in women over 50 years is well over 80 % [1] and in the symptomatic population, when combined with breast ultrasound (US), this figure increases to around 90 %. It might then be asked why another imaging modality such as breast MRI is required at all. However, it is well recognised that the sensitivity of mammography is substantially lower (around 50 %) in the mammographically dense breast, even with state of the art full field digital mammography (FFDM) [2]. Furthermore there is limited inherent contrast in mammography; many lesions are indeterminate, requiring further evaluation and biopsy; there are recognised observer limitations and it requires radiation (albeit low dose) and breast compression, which most women find very uncomfortable. Though many of these limitations are negated by high quality US, this too is operator dependent, often misses microcalcifications (the mammographic hallmark of ductal carcinoma in situ, DCIS), and also suffers from low specificity especially in the screening setting [3, 4].

On the other hand, dynamic contrast-enhanced breast MRI (DCE-MRI), the ‘bread-and-butter’ MRI technique for breast cancer detection, has a sensitivity for invasive cancers greater than 95 % in most series [5] and is the most accurate imaging technique for tumour size assessment in most circumstances [6, 7]. It can detect additional ipsilateral foci of disease in the breast known to harbour a cancer in up to as many as 25 % of cases [8], and detects synchronous contralateral occult disease with a median frequency of 4 % [9]. There are some reports suggesting that it is better than mammography for the detection of DCIS, especially more aggressive biologically relevant high grade DCIS [10]. Importantly, numerous studies have shown that DCE-MRI of the breast is a far more sensitive screening modality than FFDM or US in the detection of clinically occult breast cancer in women at greatly increased lifetime risk, especially those with BRCA mutations [11–14], with most studies showing a doubling of the cancer detection rate with breast MRI and little additional benefit from mammography.

So why is breast MRI not used more often in routine practice? In countries that have resource-limited healthcare systems, such as the UK National Health Service (NHS), timely access to MRI is a major issue, but even in resource-rich countries such as the USA, many insurance providers are refusing to reimburse breast MRI studies except in certain well defined scenarios. Most centres have seen an exponential increase in demand for breast MRI, yet to date, despite numerous studies demonstrating the superiority of breast MRI over conventional imaging in local staging, this has not translated into beneficial patient related outcomes. Specifically, the evidence suggests that use of preoperative breast MRI in patients with breast cancer results in increased mastectomy rates or larger wide local excisions [15] with, at the same time, no reduction in the incidence of positive surgical margins (necessitating re-excision) [16, 17] nor, ultimately, in ipsilateral in-breast local recurrence [18]. Similarly, though there is good evidence of stage shift as a result of the use of breast MRI for screening of high-risk women [19], it remains to be seen whether the increased cancer detection rate with breast MRI in high risk women translates into improvements in breast cancer-specific mortality [20], at least in the BRCA 1 population.

What this seeming paradox tells us is that breast MRI should be used judiciously; this is my own take on how to make it as useful as possible. There should be a very good indication for carrying out breast MRI and though space precludes a detailed exploration of the indications here, the situations in which I would either advocate or consider breast MRI for local staging are listed in Table 1. This is by no means an exhaustive list and generally, hard and fast rules are unhelpful. It is my firm view that decisions about whether or not a breast MRI is appropriate should be taken in the MDT meeting after thorough discussion. For example, take a patient with a pre-operative diagnosis of invasive lobular carcinoma (ILC). Many centres would routinely obtain a breast MR in any such patient and there is limited evidence from single centre studies that it may reduce the incidence of positive surgical margins without an increase in mastectomy rates [21]. However, it is unnecessary if conventional imaging has shown clearly that the disease is multicentric and that breast conservation is not an option. Similarly, the evidence for a higher rate of synchronous contralateral malignancy with ILC has been overstated [22] and screening of the contralateral breast is not generally indicated. On the other hand, there may be genuine uncertainty about the local extent of disease, yet if the patient’s comorbidities preclude surgical resection, there is no point in obtaining a breast MRI. Therefore, choose your indication and your patient carefully!

**Table 1:** Common Indications for Breast MRI in suspected/known breast malignancy

Adenocarcinoma of unknown primary – suspected occult breast malignancy	
Local staging – Clinical/imaging size discrepancy	
Difficulty sizing with conventional imaging – suspected multifocality	
Invasive lobular carcinoma, dense breasts	
Non-calcified DCIS	
Potential candidate for accelerated partial breast irradiation or IORT	
Response assessment (neoadjuvant chemotherapy)	
Lesion characterisation/problem solving	
Residual disease post wide local excision	
Differential diagnosis of local recurrence and treatment effects	
Screening of high risk groups (BRCA mutation carriers, previous mantle radiotherapy)	

Finally, it is incumbent on us to be aware of shifting treatment paradigms; with increasingly sophisticated oncoplastic surgical techniques, MR demonstration of multifocal disease or even segmental DCIS over as much as 6 or 7 cm need not preclude breast conservation.

### Maximising the chances of obtaining a diagnostically useful study

Once the decision to obtain a breast MRI has been made, it is important to stack the odds in your favour. I will not carry out a breast MR scan unless I have access to all relevant prior imaging, whether it be conventional mammography or MRI, and all clinical details including timing of previous biopsies or interventions, surgery or radiotherapy, and any histology results. All too often clinical details state ‘right breast cancer? extent’ – this is inadequate!

If the scan is elective, for example in the high risk screening setting, the scan should be scheduled for around day 10 of the menstrual cycle and if the patient is on HRT it may be necessary to discontinue this for 6 weeks to minimise confounding background breast parenchymal enhancement (BPE). In my experience this suggestion is often not well received, but on the other hand, this patient cohort is very highly motivated. In the patient with known breast cancer, such scheduling is not possible and note should be made of the date of the last menstrual period and hormone replacement therapy (HRT) usage.

Patient preparation is extremely important. It is impossible to overemphasise the role of sympathetic MR technicians who can talk the patient through the procedure and – critically - who are not afraid to manipulate the breast in order to optimise patient positioning within the breast coil. Cod liver oil capsules taped to the skin can be useful to mark scars or the site of the clinical abnormality. The patient must lie prone without moving for a minimum of 25 min and comfort is essential. In my unit, the patient information sheet warns patients to avoid a large meal prior to the scan as this can make lying prone for the procedure very uncomfortable. The more time spent ensuring the breasts lie centrally within the coil, with no skin folds, the better. If the MR technicians are not trained mammographers, I recommend a trip to the breast unit to gain some experience of mammography and patient positioning. Often the first scan acquired is degraded by motion artefact, so it is a good idea for this scan not to be the first of the dynamic series as it may be necessary to repeat it.

### Scan protocol

A detailed consideration of scan sequences and technical developments in sequences is outwith the scope of this article, but there are a few germane points. Though breast MRI scans can be considered to be more or less ‘out of the box’ at 1.5T, this is not true of scans at higher field strengths, when prior sequence optimisation on phantoms and healthy volunteers is essential, especially for sequences involving fat suppression (particularly diffusion weighted imaging), which can be very problematical around the breast because of susceptibility effects induced by air/soft tissue interfaces.

**Fig. 1:**
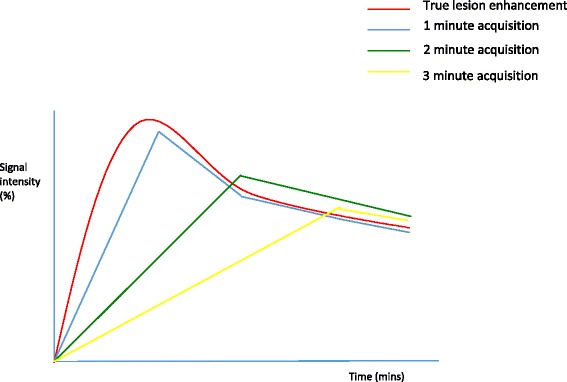
The effect of acquisition time on enhancement curves. A theoretical graphical depiction of the effect of dynamic acquisition time on apparent contrast enhancement kinetics; injection at time 0. Signal intensity (%) vs. time (minutes)

Unless there is good reason to believe that the patient will only tolerate one sequence - which should be the dynamic T1 weighted gradient echo acquisition - I commence with a high resolution axial T2 weighted TSE sequence without fat suppression (voxel size 0.9 × 0.9 × 2 mm). This is very valuable for evaluation of the morphology of masses and identification of oedema, cysts and blood products, considered in conjunction with a non fat suppressed T1 weighted 3D gradient echo sequence (often very useful for identification of marker clips). I follow this with diffusion-weighted imaging (DWI). In patients who are breast feeding or who have other contraindications to intravenous gadolinium-based contrast, the study can stop at this point and a surprising amount of information may be obtained, especially in young women with prominent fibroglandular parenchyma [23]. However, the single most important sequence remains the semi-dynamic T1 weighted gradient echo sequence, which can be 2D or 3D, with or without fat suppression. I favour an axial 3D sequence with fat suppression, voxel size 0.9 × 0.9 × 1.2 mm and acquisition time 45 s. True pharmacokinetic analysis is not possible with this sequence, but it is not necessary outside the research arena. Nonetheless, it is important to appreciate the effect of scan duration on enhancement patterns; if scan time is prolonged, rapid enhancement of a mass and washout can be missed and with a slow injection rate, peak enhancement can be dampened (Fig. 1). Conversely, information on kinetics should not be acquired at the expense of spatial resolution. For this reason I scan out to around 6 min for the dynamic series and then obtain a high resolution T1 weighted 3D gradient echo sequence with water excitation and isotropic resolution, voxel size 0.7 mm^3^, acquired in 4 minutes 30 s. This is an excellent sequence for morphology (for example, showing non-enhancing internal septations in fibroadenomata) and for those relatively rare malignancies such as low grade classical ILC, that may enhance relatively little and late.

### How I read breast MRI studies

In my institution, the medical MR physicist and I look at the scans together on the modality workstation and generate time-intensity curves from any regions of interest on the subtracted dynamic series. These are subsequently sent to PACS for reporting. If your institution has post-processing software this is very helpful, but I recommend that you ascertain from the manufactures and application specialists exactly what manipulations have been carried out on the raw data; not all softwares are equal and there is a dearth of literature on the reproducibility of results (for example, functional tumour volumes) between vendors. This may not matter for a one-off diagnostic study, but it is extremely important if, for example, one is monitoring response to neoadjuvant chemotherapy.

**Fig. 2:**
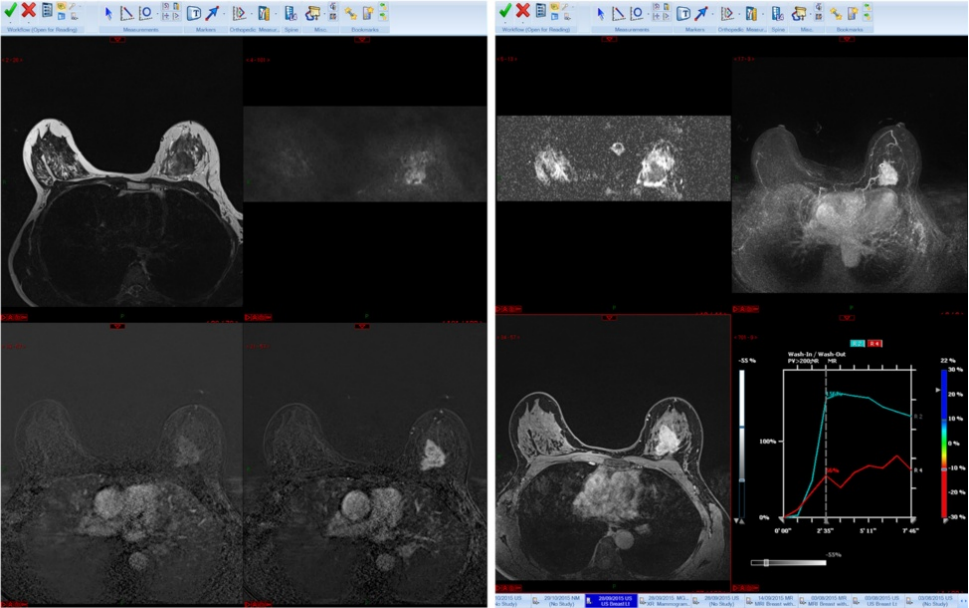
Hanging protocol. From left to right, T2 weighted, diffusion weighted series and corresponding ADC map, MIP images (*top row*). On the bottom row, from left to right, first and second subtracted series, high resolution post contrast series and post-processing

My PACS hanging protocol is shown in Fig. 2. I like to see the T2 weighted sequences, the DWI and ADC map and the axial maximum intensity projections (MIPs) across the top row. If it is the first MRI study, I will load the first and second post-contrast subtracted series, the delayed high-resolution studies and the post-processing image captures along the bottom row. If it is a follow-up study I will display the same sequences in top and bottom rows with the older examination below.

**Fig. 3:**
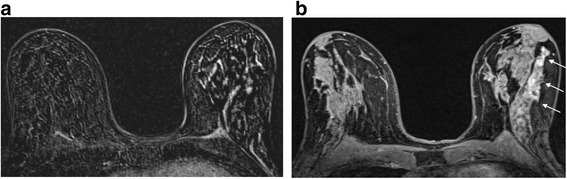
The effect of motion on subtracted images. **a** Axial post-contrast subtracted image showing severe misregistration secondary to motion in the left breast. It is not possible to identify nor gauge the extent of the known high grade DCIS in this patient. **b** Axial high resolution fat suppressed T1 weighted image post contrast. The non-mass segmental enhancement in the left breast is identifiable (*arrows*). At pathology there was 40 mm of high grade DCIS

I start by having a quick look at the MIP series to ascertain a) whether there is any significant enhancement in the breasts and b) how much movement there has been during the acquisition. Secondly, I have a look at the first and second raw and subtracted series, remembering that if there has been much motion, the subtracted series can be totally misleading. Artefactual enhancement can be recognised readily by the presence of alternating bright white and black bands and can make sizing of a lesion difficult especially if there is suspected DCIS (Fig. 3a). Here reference to the raw data and the delayed high resolution sequence can be very helpful (Fig. 3b). However it may be necessary to state in the final report that confidence in accurate sizing is limited. On the other hand, significant enhancement can be obscured and evaluation of the morphology of a mass is challenging. Postprocessing softwares generally have an algorithm for motion correction, but there are limits to what can be achieved with this, especially if motion is along the z axis.

As well as looking for the presence of significant enhancement I assess the degree of background parenchymal enhancement (BPE) around 2 min post injection of contrast. This is akin to assessing the amount of fibroglandular parenchyma on a mammogram and has the same purpose; it should indicate the level of certainty about whether or not a significant lesion is present. Just as a cancer can be obscured in the dense breast on mammography, so the presence of florid BPE can render breast MRI interpretation difficult (Additional file 1: Figure S4a and b). However, contrary to the situation with mammography, there appears to be no drop in the sensitivity with severe BPE, despite a higher rate of examinations called abnormal [24, 25]. In the latest BI-RADS lexicon, BPE is graded a to d for none/minimal through to severe; the meaningless attempt to assign a percentage figure that was present in the previous edition has, quite rightly in my view, been dropped. Care should be taken to ensure that windowing is appropriate; you should be able to ‘see in’ to the breast without making window widths so great that enhancement cannot be appreciated. Note that BPE can be asymmetrical, as shown in Additional file 1: Figure S4c in a patient who received unilateral whole breast adjuvant radiotherapy.

If abnormal enhancement is present, I next look at the T2W series for a morphological correlate. This yields useful information not only about the possible nature of a mass, but also, in the case of known cancers, the likely imaging phenotype. For example, Fig. 2 demonstrates the typical MR appearances of a grade 3, hormone receptor negative cancer. The T2W scan can also enable one to dismiss small foci of enhancement and can be very useful to confirm, for example, that an ovoid focus of enhancement with washout is in fact an intramammary lymph node. Linking the various series together makes this process straightforward.

**Fig. 4:**
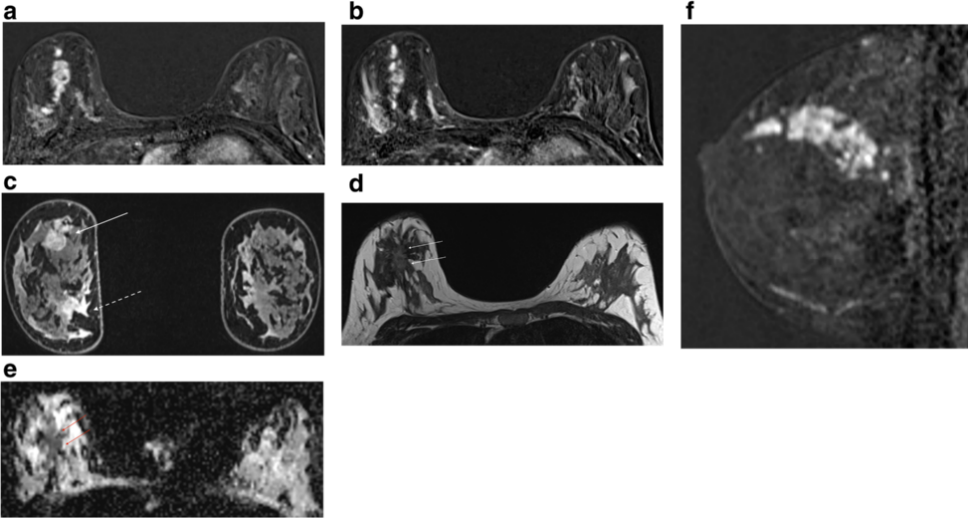
Multifocal carcinoma in a patient with florid BPE (same patient as in Additional file 1: Figure S4b). First (**a**) and second (**b**) post-contrast subtractions showing diminished tumour to background contrast in the second acquisition. **c** Delayed high resolution fat suppressed T1W image showing tumour at 12 o’clock (*solid arrow*) and florid BPE especially at four o’clock (*dashed arrow*). Note similar signal intensities in the two areas. Axial T2W (**d**) and corresponding ADC map (**e**) show subtle T2 hyperintense tumour and obvious restriction of diffusion in the mass. Note similarity of distribution of restricted diffusion to enhancing tumour in (**a**). Extent of tumour for treatment planning is well depicted in the sagittal reconstruction of the first post-contrast subtraction (**f**)

**Fig. 5:**
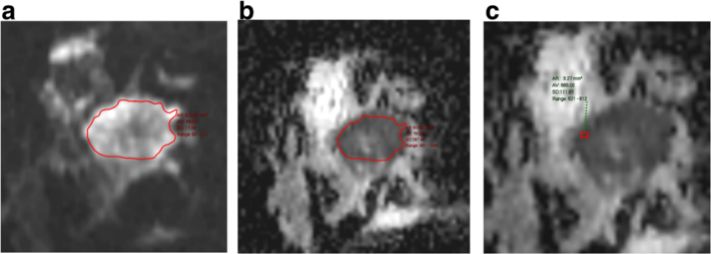
ADC measurement in a grade 3 triple negative breast cancer with some central necrosis. **a** b850 image (**b**) whole tumour ADC (**c**) ‘hot spot’ or ADC_min_ which is substantially lower

**Fig. 6:**
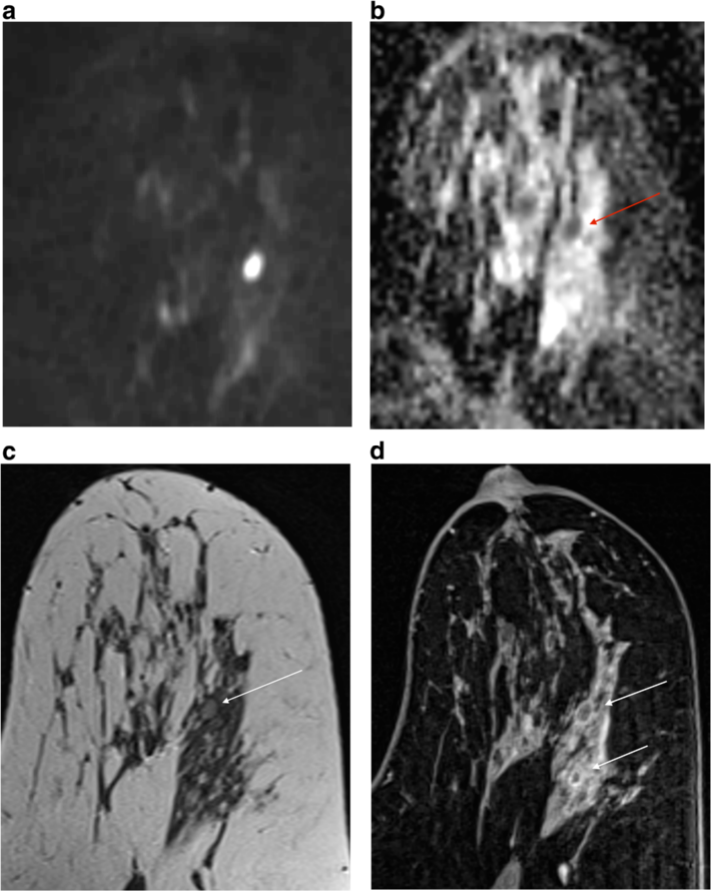
Cystic benign change. Axial b850 image (**a**), corresponding ADC map (**b**), T2 weighted image (**c**) and post-contrast T1 weighted image demonstrating restriction of diffusion in a proteinaceous cyst (**d**). There is an ovoid lesion with high b850 signal (**a**) and restricted diffusion (*arrowed*) (**b**). There is intermediate signal within it on T2W imaging (**c**) but the high resolution post-contrast sequence shows a small amount of enhancement around a cyst, with other cysts elsewhere (**d**)

If there is a T2 correlate I routinely look at the DWI and corresponding apparent diffusion coefficient (ADC) map. I use b values of 50 and 850. Whilst I accept that DWI is not necessary for the interpretation of breast MRI scans, there are occasions when it can be very helpful, provided the series is of acceptable quality. In the presence of a known cancer and florid BPE, working out exactly what is malignant and what is not can be very difficult and it is here that the DWI can be helpful [26] (Fig. 4). However, if you are trying to evaluate a 7 mm mass or non-mass enhancement in a fatty breast, and the DWI slices are 4 mm thick with a 2 mm gap and poor fat suppression, it is probably not going to yield any useful information. It may be enough merely to ‘eyeball’ the ADC map to establish whether there is restriction of diffusion, but I generally copy and paste a ROI from the high b value image to the ADC map. A catch to be aware of is that certain high grade tumours, particularly triple negative basal phenotype cancers, commonly have areas of necrosis and may therefore have high whole-lesion ADC values [27]. Use of a small ROI may be more discriminatory (Fig. 5). On the other hand, proteinaceous cysts may exhibit increased signal at high b values, a low ADC and only intermediate T2 signal. In instances like this a quick glance at the DCE series enables the correct diagnosis (Fig. 6). It is also important to be aware of the presence of any marker clips or staples, where susceptibility artefacts preclude useful ADC measurements.

**Fig. 7:**
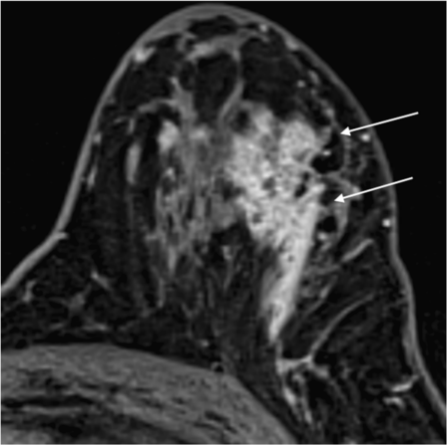
Clustered ring enhancement in a patient with extensive DCIS

The next step is kinetic and morphological analysis of any enhancement using the BI-RADS lexicon. In the latest edition, published in 2013 [28], the descriptors have been simplified and aligned with those in the mammography and ultrasound sections. It is possible to download a free pdf from the ACR BI-RADS website that summarises the MRI lexicon and I recommend having this to hand when reporting if you are not used to the descriptors. The main changes in the lexicon are summarised in Table 2. Descriptors that were infrequently used (such as central enhancement and enhancing internal septations) have been removed. Non-mass *like* enhancement becomes non-mass enhancement and the terms ‘reticular/dendritic’ and ‘stippled’ used to describe it have also been removed, as they were used infrequently and stippled enhancement is recognised as a normal type of BPE. One addition is the term ‘clustered ring’ to describe a form of non-mass enhancement often associated with DCIS, as shown in Fig. 7.

**Table 2:** A summary of key changes in the BI-RADS MRI lexicon

Feature	2013 BI-RADS Atlas
Breast composition	a (fatty)through to d (extreme FGT)
BPE level	Minimal, mild, moderate, marked
BPE distribution	Symmetric or asymmetric
Focus	Removed from BPE section
Mass shape	‘lobular’ removed: oval, round or irregular only
Mass margin	‘smooth’ removed: circumscribed, irregular or spiculated only
Mass internal enhancement	‘Enhancing internal septations’ and ‘central enhancement’ removed
Non-mass enhancement (nme)	Non-mass *like* removed
Nme distribution	‘ductal’ removed
Nme internal enhancement characteristics	‘Stippled/punctate’ removed (a normal pattern of BPE)
	‘reticular/dendritic’ removed
	‘clustered ring’ added
Intramammary lymph node	New addition as separate feature
Skin lesion	New addition as separate feature
Associated findings	Skin invasion: new descriptors (‘direct invasion’, ‘inflammatory cancer’)
	Oedema: removed
	‘Lymphadenopathy’: removed. Now termed ‘axillary adenopathy’
	‘Chest wall invasion’ added; separate from pectoral muscle invasion
	‘Nipple retraction’ removed
Non-enhancing findings	Ductal precontrast high signal on T1W added
	Cyst added
	Postoperative collections (haematoma/seroma) added
	Post therapy skin/trabecular thickening added
	Architectural distortion added
Fat containing lesions	New section (includes fat necrosis, lymph nodes, hamartomas etc.)
Kinetic curve assessment	New section: initial phase (slow, medium, fast), delayed phase (persistent, plateau, washout)
Implants	New section: includes material and type, location, evidence of rupture, abnormal implant contour, signs of intracapsular rupture; extracapsular silicone (breast or lymph nodes), water droplets or peri-implant fluid

**Fig. 8:**
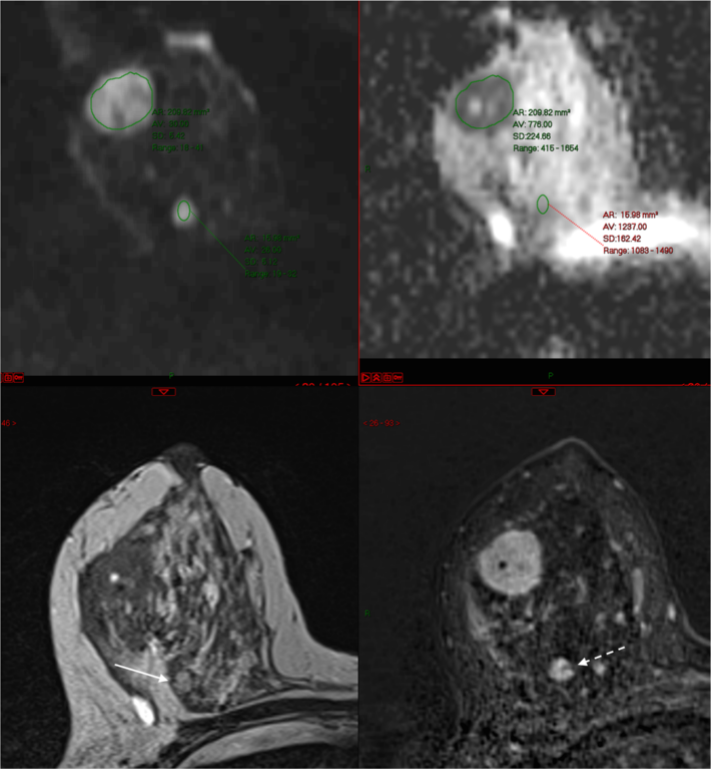
Multiparametric breast MRI. From top left to bottom right: DWI (b850), ADC map, T2W image and T1W post-contrast subtracted image. There is an obvious carcinoma in the upper outer quadrant of the right breast. An unexpected second rounded enhancing mass deep in the right breast is slightly hyperintense on the T2 weighted image (*arrow*) and has high signal on the b850 image, but there is no restriction of diffusion. Notice also a non-enhancing internal septation (*dashed arrow*). Biopsy-proven fibroadenoma

When evaluating mass or non-mass enhancement I prefer to link the relevant series, as shown in Fig. 8, so that multiparametric assessment of any finding is facilitated. After I have made a morphological assessment using the BI-RADS descriptors, kinetic assessment follows. All of the major MRI manufacturers have their own analysis packages which produce colour overlays on the dynamic series; generally speaking a lesion that is bright red is one that is enhancing rapidly, above a certain percentage threshold. These overlays are useful in drawing one’s attention to areas of enhancement where time-intensity curves should be drawn. I tend to move a small region of interest around looking for the ‘worst’ curve; that is, rapid enhancement with washout (type 3 curves). Often washout can be easily appreciated from the MIP series, but of course if there is any motion during the dynamic series (a frequent occurrence) it will not be possible to generate meaningful time-intensity curves unless there is very good motion correction (Fig. 3). It is for this reason that I always evaluate the raw data as well as the subtracted series. On the other hand, without the subtracted series, high T1 signal in the ducts could be wrongly interpreted as segmental enhancement rather than the presence of proteinaceous fluid as occurs with duct ectasia.

**Fig. 9:**
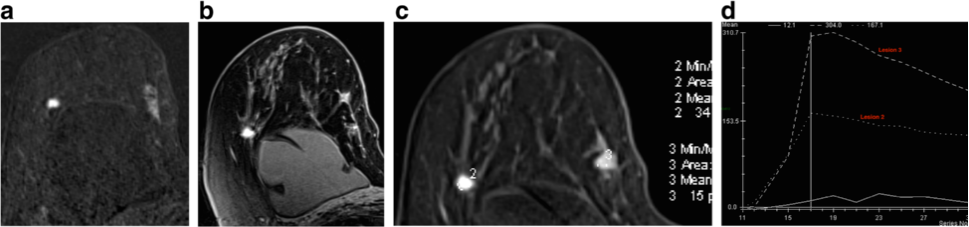
Patient with known grade 1 classical invasive lobular carcinoma in the upper inner quadrant of the right breast. MRI was indicated as the patient had breast implants and the breasts were difficult to assess with conventional imaging. Unexpected finding of a second carcinoma in the upper outer quadrant. **a** Post contrast subtracted image, (**b**) high resolution delayed post contrast image, (**c**) regions of interest and (**d**) time-intensity curves. Note different morphology and kinetics of the two lesions; lesion 3 in the lateral breast was a hormone receptor positive grade 3 invasive ductal carcinoma

As a general principle it is most helpful to look at the early post-contrast subtractions to differentiate between malignancy and florid BPE (Fig. 4). During later acquisitions, there may be very little tumour-to-background contrast because of washout from the malignancy and persistent enhancement of BPE. Often, unsuspected foci of ipsilateral malignancy tend to have the same enhancement characteristics as the index lesion – though beware instances of two different immunophenotypes within the same breast (Fig. 9).

Certain key measurements need to be documented in the report. Not only should the size of a lesion be given (in three planes), but also the quadrant, clock face position and distance from the nipple. Generation of ‘route maps’ in the appropriate plane are helpful, not only for the surgeon but also for the unfortunate radiologist or sonographer who may have to do a second-look targeted ultrasound. If there are small satellite lesions within a few mm of a known cancer I tend to include these in the overall dimensions, but if the MRI demonstrates further lesions that were not expected, it is essential to document their location and their relationship with the index lesion; again, reformatted route maps are very useful.

Before I finish reviewing a study I have a mental checklist of review areas. The axilla of a cancer–bearing breast must be examined carefully and if there is obviously a heavy nodal burden the axillary apex and supraclavicular fossa should be reviewed. It is easy to miss enlarged lymph nodes in the internal mammary chain, an important observation as it may affect radiotherapy planning. Satisfaction of search is important; whereas it is hard to overlook an enhancing lesion in the contralateral breast, it is very easy to overlook small bone metastases, liver lesions or lung nodules. All of these are commoner with a large primary tumour (T3 or 4) and N2 nodal disease and there is a particularly high incidence with inflammatory breast cancer, a condition that is readily apparent with breast MRI.

### Assigning a BI-RADS category

In MRI, BI-RADS 1 and 2 lesions have no probability of malignancy. It is a question of preference whether to mention entities such as cysts, old scars or obvious fibroadenomas; if you do, you are bound to call these BI-RADS 2. I try always to minimise the number of category 3 (probably benign) lesions as these are a bit of an unknown quantity in breast MRI. Though in some retrospective series, with variable follow-up and histopathological correlation, the rate of malignancy is low (around 2 %) [29, 30], in other series it is around 4 % [31]. The presence of a T2 correlate, any restriction of diffusion and the size of the lesion can be helpful. There is evidence that the rate of BI-RADS 3 categorisation decreases with experience and with maturation of a screening programme; ideally the rate should be well under 10 % and preferably nearer 3 %. BI-RADS 4 lesions have a probability of malignancy of between 5–95 % and thus constitute a bit of a dumping ground; but importantly, these lesions should not be left alone. It is here that correlation with non-contrast MRI and conventional imaging can be really helpful. For example, if a small mass has features of a lymph node, the presence of washout does not matter; this is a category 2 lesion. Fat necrosis can appear highly suspicious, with spiculate masses and washout kinetics, but the diagnosis should be apparent from evaluation not only of the non-contrast images but also conventional imaging.

In considering categorisation, morphology and kinetics should be considered together, but morphology is the most important feature. Certain carcinomas may have type 1 curves (especially classical ILC), but the morphology is usually highly suspicious. Kinetic analysis may not be possible at all with linear non-mass enhancement as seen with DCIS, especially if there has been any movement. Conversely, some myxoid fibroadenomata may have washout curves; here the T2 correlate and DWI signal is very useful. The morphological feature with highest PPV for malignancy is spiculation, followed by irregular shape or margin, and heterogeneous or rim enhancement [32–34]. Clumped nodular and clustered ring enhancement are the features of non-mass enhancement with the highest PPV for malignancy [31, 35]. On the other hand, round or oval, smooth non-enhancing masses or masses with non-enhancing internal septations are virtually never malignant. Finally, BI-RADS 5 lesions have a greater than 95 % chance of malignancy. In the US, a known biopsy proven carcinoma is category 6, though this category tends not to be used in the UK. I can think of only a handful of occasions when I struggled to assign this category to a known invasive carcinoma; two were mucinous carcinomas, and the rest were very small screen-detected ILC that barely enhanced at all. On the other hand, though some authors report an exceptionally high sensitivity of MRI for DCIS, especially high grade [10], it is not uncommon to miss intermediate and low grade DCIS especially if the scans are poor quality.

It is important to remember that even in the presence of a known malignancy, multiple small enhancing foci are nearly always benign [31] and I try not to overcall these. Otherwise there is the risk of overstaging, especially with invasive lobular carcinoma. Patients undergoing local staging will usually have had image guided biopsy, which can results in peritumoural stranding, and (usually) mild enhancement – this should not be mistaken for the presence of an associated extensive DCIS.

### The report and management recommendations

As emphasised in the excellent introductory overview on how to read cancer imaging studies by Professor Hicks, probably the single most important factor in the issuing of a helpful report is a thorough understanding of the precise clinical question and of the factors that will influence the treatment plan. Above all, keep a sense of perspective – when a patient has a grade 3, triple negative breast cancer that is shown on MRI to be locally advanced (T4) with obvious extensive nodal involvement, the presence of a small focus of non-mass enhancement that is indeterminate (BI-RADS MRI 3) in the contralateral breast is to all intents and purposes irrelevant. The same is true of a similar focus in a different quadrant of the same breast since it is highly likely that the patient will ultimately require mastectomy. On the other hand, the decision to go for mastectomy should not be made on the basis of a second lesion without histological confirmation.

By and large, a BIRADS MRI 3 mass less than 5 mm or a focal non-mass enhancement under 10 mm does not need further evaluation [36]. Thus, recommending a second look ultrasound or even MRI-guided biopsy may not be necessary, though in the US this would generally mandate short interval (6 month) follow-up. It helps to think about what you will do if you cannot find the lesion on ultrasound; is there sufficient concern that MRI guided biopsy would then be considered? If so, it should probably be a category 4 lesion.

For BI-RADS 3 lesions that are larger than 5 mm (masses) or 10 mm (nme) I would generally perform second look ultrasound if it will influence management. Reassuringly, the incidence of malignancy in lesions without a second look ultrasound correlate is relatively low (though variable) [37, 38], but attention to scan technique is critical. For BI-RADS 4 lesions, further evaluation is always indicated. With second look ultrasound, careful attention must be paid to altered spatial relationships. The sonographic correlates of the MRI lesion are often very subtle [39] and if anything is seen that might correspond to the lesion, it should be biopsied and a marker clip inserted. I have found two techniques to be helpful in this regard; firstly, the use of shear wave elastography to help identify subtle lesions and secondly, the use of ultrasound guided vacuum assisted biopsy. This is particularly helpful in cases of segmental nme, where the location of the abnormality is known. This will often result in definitive histology. Failing that, MRI guided biopsy is necessary and this should be done in a timely fashion so that there are no delays in the patient pathway.

**Fig. 10:**
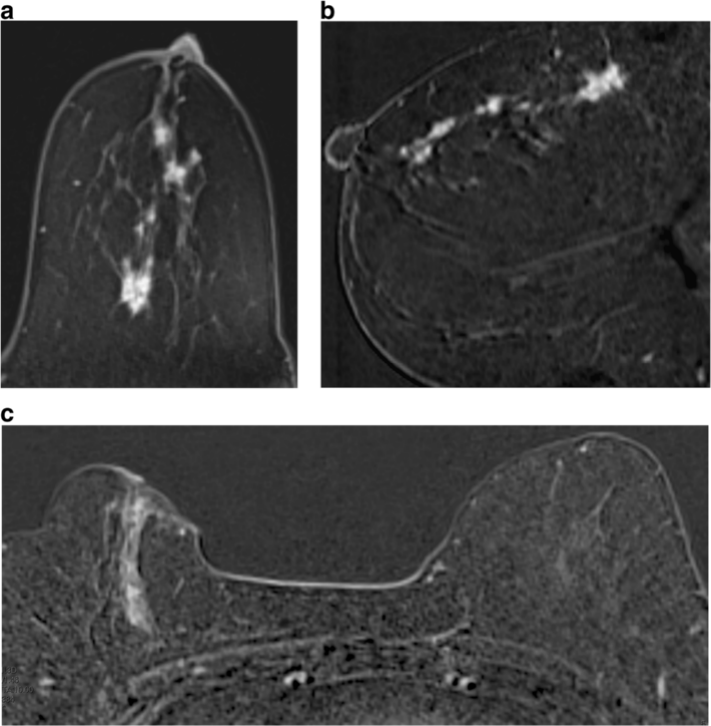
Two different patients with DCIS. **a**, **b** There is segmental clumped nodular enhancement over at least 6 cm at 12 o’clock, extending to the nipple. **c** There is segmental linear non mass enhancement at two o’clock in the right upper inner quadrant over 4.5 cm. Breast conservation was possible in the first case but not in the second

For cases of known cancer staging, I give a T stage where possible. However, it is worth remembering that surgical management of a cancer depends not only on the absolute size of the lesion in relation to the size of the breast, but also on the site of the abnormality. A 3 cm lesion can often be treated by wide local excision if it is situated in the upper outer quadrant of a large breast; but this will not be the case if the lesion lies in the upper inner quadrant. Similarly, the orientation of the malignancy has a significant impact on the treatment options. A lesion that is oriented radially towards the nipple can often be resected even if it is over 5 cm in length (Fig. 10); whereas if the maximal diameter is in the coronal plane, breast conservation will rarely be possible even with oncoplastic techniques. Finally, if the patient obviously has more than four lymph nodes that are involved by metastatic disease, I will recommend whole body staging if this has not already been carried out.

There are instances where I pursue indeterminate findings much more aggressively; namely in the case of women with BRCA mutations undergoing screening MRI, especially if there is a known or suspected BRCA 1 mutation. BRCA 1 cancers tend to grow extremely rapidly and have a distinct phenotype; they often appear benign, being rounded and relatively well defined [40]. Washout kinetics need not be present when they are small and they can look remarkably like fibroadenomas. This is one instance in which I may pursue masses under 5 mm in size, as these tumours have a very fast doubling rate. If no lesion is found on second look ultrasound I would then advise either very short interval follow-up or MR guided biopsy. The management approach I use is summarised in Table 3.

**Table 3:** A summary of management recommendations

Cases for second look ultrasound	
Normal mammogram	
Indeterminate or suspicious masses >5 mm	
Areas of focal nme >10 mm	
Recommend what should be done if no ultrasound correlate can be identified	
Cases for MRI guided biopsy	
Normal mammogram and second look ultrasound	
Indeterminate or suspicious masses >5 mm	
Areas of focal nme >10 mm	
Cases for follow-up MRI (screening)	
Suspicious mass <5 mm or nme <10 mm: follow up at 6/12 (BRCA 1) or 12/12	
Inconclusive biopsy: follow up at 6/12	

## Conclusions

Breast MRI is a remarkably powerful tool but if we are to do no harm, the onus is on us to appreciate the limitations of the technique and to issue a clear and concise report that details the level of concern and the actions, if any, that need to be taken. I am a great believer in brevity when it comes to reports; I want my reports to be read! Therefore I tend not to exhaustively list all the scan parameters and all the normal/benign findings. If resources permit double-reporting, this is highly desirable especially when a service is being introduced. Indeed, in the UK it is a quality requirement for high risk family history scans, which are carried out under aegis of the NHS breast screening programme. Failing that, adoption of a standardised approach to reporting, such as the one I have discussed above, minimises the likelihood of errors or omissions. In summary:

- Have a thorough understanding of the strengths and weaknesses of breast MRI
- Have a good indication and choose your patients carefully
- Ensure the patient is well prepared
- Understand the precise clinical question and the findings that will alter treatment
- Use recognised descriptors in your report
- Describe precisely where lesions are in relation to landmarks such as the nipple; give a T stage
- Check all nodal stations carefully including apical and internal mammary lymph nodes
- Remember satisfaction of search; check the other breast!
- Check for extramammary findings (lungs, bone visualised liver)
- Give a concise report with a final assessment score and a clear management recommendation

## Abbreviations

ADC, apparent diffusion coefficient; BI-RADS, breast imaging and reporting data system; BPE, background parenchymal enhancement; DCE-MRI, dynamic contrast-enhanced magnetic resonance imaging; DCIS, ductal carcinoma in situ; DWI, diffusion weighted imaging; FFDM, full field digital mammography; HRT, hormone replacement therapy; ILC, invasive lobular carcinoma; MIP, maximum intensity projection; NHS, National Health Service; TSE, turbo spin-echo; US, ultrasound

## Additional file

Additional file 1: Figure S4. Background parenchymal enhancement. **a** Axial post contrast subtracted image; minimal BPE in the right breast and a large enhancing cancer in the left upper inner quadrant (same patient as in Fig. 2). **b** Axial MIP series post intravenous contrast in a patient with known right breast cancer, for staging MRI. Severe BPE. **c** MIP image in a patient who has previously had radiotherapy to the right breast. Note absence of BPE in the right breast compared to the left. (ZIP 4210 kb)
