# Supplementary figures and images for: How I report breast magnetic resonance imaging studies for breast cancer staging and screening
Source: Cancer Imaging. 2016 Jul 25;16:17. doi: 10.1186/s40644-016-0078-0 (PMC4960688; doi:10.1186/s40644-016-0078-0)

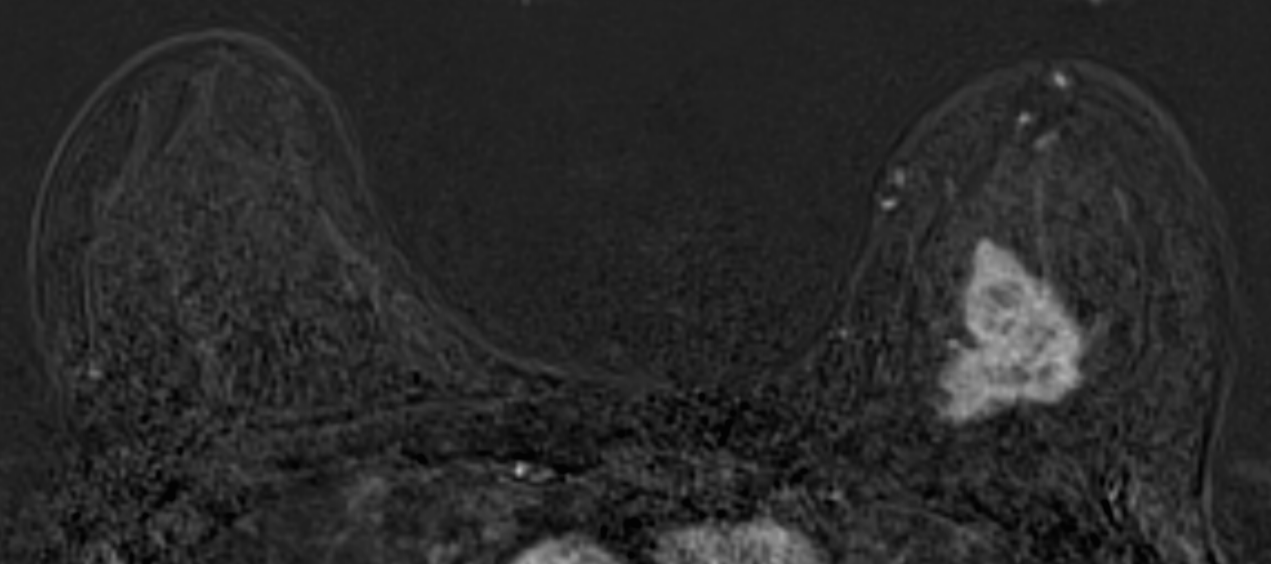

Supplement: Additional file 1: Figure S4. — Background parenchymal enhancement. a Axial post contrast subtracted image; minimal BPE in the right breast and a large enhancing cancer in the left upper inner quadrant (same patient as in Fig. 2). b Axial MIP series post intravenous contrast in a patient with known right breast cancer, for staging MRI. Severe BPE. c MIP image in a patient who has previously had radiotherapy to the right breast. Note absence of BPE in the right breast compared to the left. (ZIP 4210 kb) [file 40644_2016_78_MOESM1_ESM.zip › Fig 4 a.tiff]

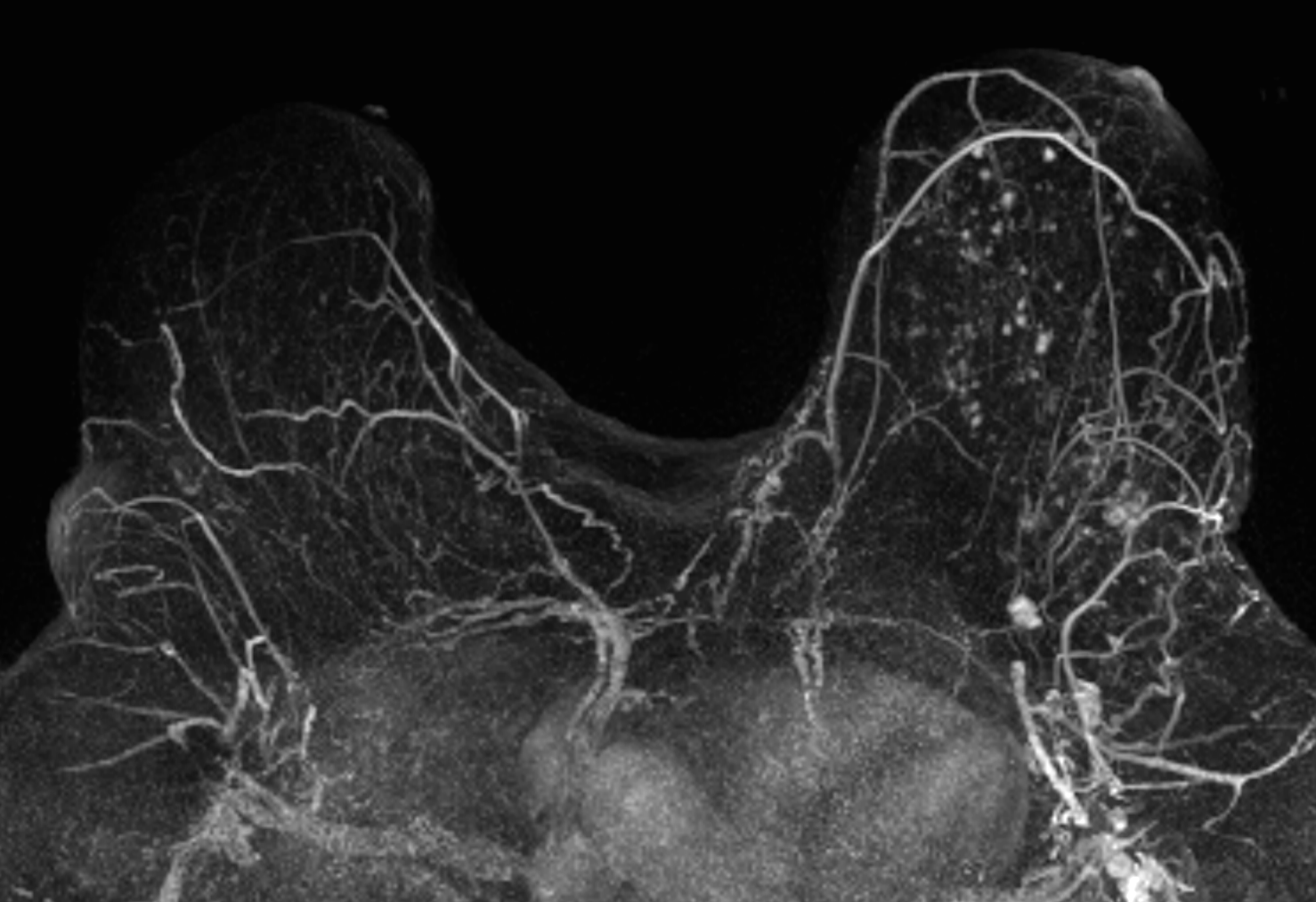

Supplement: Additional file 1: Figure S4. — Background parenchymal enhancement. a Axial post contrast subtracted image; minimal BPE in the right breast and a large enhancing cancer in the left upper inner quadrant (same patient as in Fig. 2). b Axial MIP series post intravenous contrast in a patient with known right breast cancer, for staging MRI. Severe BPE. c MIP image in a patient who has previously had radiotherapy to the right breast. Note absence of BPE in the right breast compared to the left. (ZIP 4210 kb) [file 40644_2016_78_MOESM1_ESM.zip › Fig 4 c.tiff]
